# Lipid-Based Nanoparticles: Application and Recent Advances in Cancer Treatment

**DOI:** 10.3390/nano9040638

**Published:** 2019-04-19

**Authors:** Beatriz García-Pinel, Cristina Porras-Alcalá, Alicia Ortega-Rodríguez, Francisco Sarabia, Jose Prados, Consolación Melguizo, Juan M. López-Romero

**Affiliations:** 1 Institute of Biopathology and Regenerative Medicine (IBIMER), Biomedical Research Center (CIBM), University of Granada, 18100 Granada, Spain; beatrizgarnel@correo.ugr.es (B.G.-P.); melguizo@ugr.es (C.M.); 2 Instituto de Investigación Biosanitaria ibs.GRANADA, 18012 Granada, Spain; 3 Department of Anatomy and Embryology, Faculty of Medicine, University of Granada, 18016 Granada, Spain; 4 Department of Organic Chemistry, Faculty of Sciences. University of Málaga, 29071 Málaga, Spain; cristinaalcala@uma.es (C.P.-A.); aliorterodri@uma.es (A.O.-R.); frsarabia@uma.es (F.S.); jmromero@uma.es (J.M.L.-R.)

**Keywords:** lipid-based nanoparticles, cancer, drug release, drug resistance, clinical trials

## Abstract

Many therapeutically active molecules are non-soluble in aqueous systems, chemically and biologically fragile or present severe side effects. Lipid-based nanoparticle (LBNP) systems represent one of the most promising colloidal carriers for bioactive organic molecules. Their current application in oncology has revolutionized cancer treatment by improving the antitumor activity of several chemotherapeutic agents. LBNPs advantages include high temporal and thermal stability, high loading capacity, ease of preparation, low production costs, and large-scale industrial production since they can be prepared from natural sources. Moreover, the association of chemotherapeutic agents with lipid nanoparticles reduces active therapeutic dose and toxicity, decreases drug resistance and increases drug levels in tumor tissue by decreasing them in healthy tissue. LBNPs have been extensively assayed in in vitro cancer therapy but also in vivo, with promising results in some clinical trials. This review summarizes the types of LBNPs that have been developed in recent years and the main results when applied in cancer treatment, including essential assays in patients.

## 1. Introduction

Cancer nanotechnology as a way of anticancer drug delivery has been developed as a promising cancer treatment [1]. Nanoparticles present sizes between 1 and 1000 nm and they improve drug bioavailability as well as the selectivity of the anticancer drug [2]. Among many nanoformulations used in oncology, we highlight those based on lipid formulations, since great advances have been made in recent years with respect to preparation and alternative compositions [3].

Lipid-based nanoparticles (LBNPs) such as liposomes, solid lipid nanoparticles (SLN) and nanostructured lipid carriers (NLC) have received great attention in drug discovery and cancer treatment. These nanoparticles can transport hydrophobic and hydrophilic molecules, display very low or no toxicity, and increase the time of drug action by means of a prolonged half-life and a controlled release of the drug [4]. Lipid nanosystems can include chemical modifications to avoid the detection by the immune system (gangliosides or polyethylene glycol (PEG)) or to improve the solubility of the drug. In addition, they can be prepared in formulations sensitive to the pH in order to promote drug release in an acid environment, and can also be associated with antibodies that recognize tumor cells or their receptors (such as folic acid (FoA)) [5]. Nanodrugs can also be used in combination with other therapeutic strategies to improve the response of patients.

Many antitumor agents, such as cisplatin, irinotecan (IRI), paclitaxel (PTX), doxorubicin (DOX) oxaliplatin, daunorubicin, cytarabine or vincristine, have been studied in nanoformulations, and some of them have been analyzed in clinical trials and/or are commercially available for clinical use in patients [6]. In fact, Doxil^®^, a liposome formulation encapsulating DOX, was one of the first anticancer drug nanosystems commercially used.

This review highlights the main contributions to LBNPs that have been developed in recent years and applied in cancer treatment, including essential assays in patients.

## 2. Lipid-Based Nanoparticles

### 2.1. Liposomes

Liposomes are the most studied delivery systems due to the biocompatibility and biodegradability that they present. The main components of these nanoparticles are phospholipids, which are organized in a bilayer structure due to their amphipathic properties. In presence of water, they form vesicles, improving the solubility and stability of anticancer drugs once they are loaded into their structure. They are capable of encapsulating either hydrophobic or hydrophilic drugs [7]. In addition to phospholipids, other compounds can be added to their formulations, such as cholesterol, which decreases the fluidity of the nanoparticle and increases the permeability of hydrophobic drugs through the bilayer membrane, improving the stability of these nanoparticles in blood [7]. Cholesterol-modified liposomes may present a multiple bilayer with sizes from 0.5 nm to 10 nm, named Multilaminar Vesicles (MLVs); a single bilayer with sizes above 100 nm, named Large Unilamellar Vesicles (LUVs); and intermediate sizes (10–100 nm), which are called Small Unilamellar Vesicles (SUVs) [7].

It is well known that there are two general ways to achieve nanocarrier vectorization. One of them is passive targeting, which occurs when liposomes only enter the tumor cell by molecular movement through the cellular membrane. The other is active targeting, which involves structurally modified liposomes holding antibodies that recognize tumor cells [8]. A third method can be considered for liposomes, which is when they have been prepared with stimulus-sensitive structures. Temperature, pH or magnetic fields are parameters that can be modulated for the controlled delivery of an anticancer drug by using an external trigger [9].

**Figure 1:**
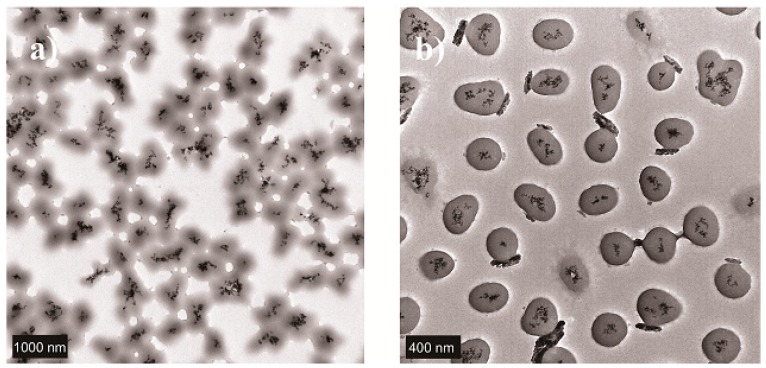
(**a**) 1000 nm scale TEM-image of Fe_3_O_4_ magnetic nanoparticles. In the 400 nm scale TEM-image (**b**) magnetite cores can be clearly observed as black spots.

The synthesis and development of new liposomes have been extensively studied in recent years. In fact, Fe_3_O_4_ cores are increasingly being used to functionalize different types of nanoparticles (Figure 1). In 2014, chemotherapy and hyperthermia treatment were combined by using liposome-encapsulated DOX, which included citric acid-coated magnetic nanoparticles [10]. Also in 2014, DOX was co-encapsulated with Magnevist^®^, a contrast agent; both actives were included in a liposome modified with amphiphilic hyaluronic acid and cholesterol [11]. Moreover, ultrasound-sensitive liposomes were developed for DOX encapsulation [12], such as in the case of the thermo-sensitive polymer *poly*(NIPMAM-co-NIPAM), which can be degraded by sonication, leading to drug release. On the other hand, liposomes modified with PEG and anacardic acid have been developed, and these were employed to encapsulate docetaxel, improving the stability of this anticancer drug [13].

Yan et al. [14] developed a pH-responsive liposome coated with a glycol derivative chitosan. These liposomes presented terminal amine groups, which conferred a superficial negative charge to interact with the positive charge present in the acidic extracellular tumor media. DOX was loaded into this liposome, and a remarkable improvement of its anticancer efficacy was observed [14]. Ramadass et al. [15] co-encapsulated epigallocatechin gallate (a type of catechin) and PTX in this type of liposome for the treatment of lung cancer. In vivo studies showed that this liposome-based treatment was more effective compared with treatments with free drugs [15]. Ali et al. [16] synthetized a PEGylated liposome, whereby they encapsulated doxorrubicin hydrochloride instead of DOX. This new liposome-based formulation of DOX proved to be safe and effective against ovarian cancer [16]. In this year, another PEGylated liposome was developed by Meng et al. [17] with a size of 50 nm to co-encapsulate two different anticancer drugs, resveratrol and PTX, as a novel delivery system to combat the resistance of cancer. On the other hand, Xiao et al. [18] developed a liposome modified with gadolinium to improve the solubility of sorafenib, an anticancer drug with low solubility in water. Additionally, they studied this system of anticancer drug delivery in vivo as well as in vitro, demonstrating that this liposome could also be applied as a contrast agent [18].

Gogoi et al. [8] explored novel magnetic liposomes. To this end, they co-encapsulated dextran, La_0.75_Sr_0.25_MnO_3_ and iron oxide to combine self-controlled hyperthermia and chemotherapy. PTX was encapsulated in this type of hybrid liposome. In vitro and in vivo biological studies demonstrated the biocompatibility and therapeutic efficacy of this new magnetic liposome [8]. In 2018, various liposomes were developed to encapsulate different anticancer drugs. For example, Sushant Lakkadwala et al. [19] designed a liposome modified with penetrating peptides and transferrin to encapsulate 5-fluorouracil (5-FU) for the treatment of brain cancer. The studies in vivo showed that this dual-functionalized liposome could transport the anticancer drug through the brain barrier and delivery 5-FU to tumor cell [19]. In another study, Deshpande et al. [20] developed a modified liposome with PEG and arginine-rich cell-penetrating peptides. Furthermore, this liposome presented a specific ligand to interact with the tumor. In this liposome, DOX was encapsulated improving delivery and efficacy in vivo and reducing citoxicity in vitro [20]. DOX was also co-encapsulated with curcumin (CUR) in a long-circulating liposome by Sesarman A et al. [21]. Studies in vivo of this novel delivery system showed that antitumor activity increased on C26 colon carcinoma [21]. A noteworthy case was the liposomes developed by Tian et al. [22], who encapsulated PTX in a modified liposome with hyaluronic acid, a molecule biodegradable and biocompatible which interacts with a specific ligand present in the tumor cells.

### 2.2. Solid Lipid Nanoparticles (SLN)

**Figure 2:**
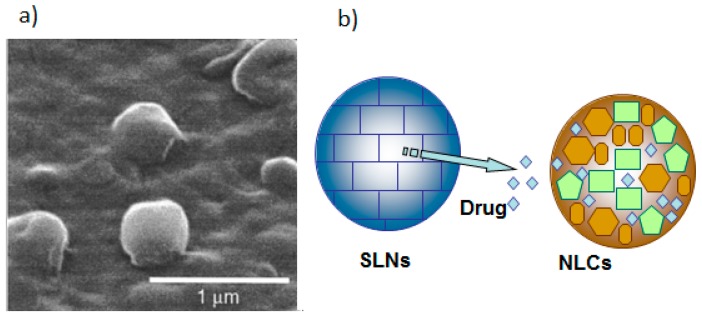
(**a**) SLNs SEM image. (**b**) In comparison with NLCs, SLNs high drug-loading capacity and drug expulsion due to the crystallization process during the storage conditions.

SLNs represent a relatively new colloidal drug delivery system, composed of physiological lipids that remain in a solid state at both room and body temperature. These particles are in the size range of 50–1000 nm. The solid lipid used forms a matrix material for drug encapsulation and include mono-, di- or triglycerides, fatty acids and complex glyceride mixtures. This matrix is stabilized by a mixture of surfactants or polymers. SLNs have significant advantages, such as site-specific targeting, physical stability over a long period, possibility of controlled release of both lipophilic and hydrophilic drugs, protection of labile drugs, low cost, ease of preparation and nontoxic. Furthermore, in reference to toxicity, SLNs have exceptionally low toxicity effects against human granulocytes. All these outstanding advantages make them an important candidate for drug delivery systems [23]. In contrast, SLNs present some disadvantages, such as moderate drug-loading capacity and drug expulsion due to the crystallization process under storage conditions (Figure 2) [24].

Given their unique properties, SLNs have been extensively studied for drug delivery of active anticancer compounds, incorporating them into the SLN formulation in order to improve the oral bioavailability of drugs, protect labile anticancer drugs, and also to reduce side effects by decreasing the dosage by effective targeting to the site of action. The main contributions reported in the last year show that SLNs can be used for encapsulated anticancer drugs. For example, in 2018, Pindiprolu et al. [25] developed niclosamide-loaded SLNs, which improved cell uptake and anticancer efficacy against triple negative breast cancer cells (TNBC). Also in 2018, the group of Eskiler [26] formulated talazoparib loaded onto SLNs, improving its therapeutic index against TNBC cells. This improvement was as a consequence of minimizing toxicity and overcoming homologous recombination (HR)-mediated resistance. Other research groups, like [27], prepared magnetic SLNs formulation of PTX in order to control temperature-dependent drug release, using magnetic hyperthermia to trigger controlled PTX release from magnetic SLNs. In another example, citral- and geraniol-loaded SLNs showed anti-inflammatory activity in the RAW 264.7 cell line, having enhanced capacity to inhibit NO production [28]. Also of interest were the resveratrol-loaded SLNs, which were used to treat human breast cancer cells [29]. In this case, the authors found that resveratrol SLNs showed superior ability in inhibiting the cell proliferation compared to free resveratrol. Also, they exhibited much stronger inhibitory effects on the invasion and migration of cells, suggesting that resveratrol-SLN has great potential for breast cancer (BreC) treatment. In another study, it was demonstrated that SLNs can also be used as a vehicle to increase floxuridine efficacy in cancer therapy, due to an improved cellular uptake [30]. In this case, loading the lipophilic prodrug floxuridine—an effective anticancer drug with high potency—into SLNs could be a solution to the low efficiency of cellular uptake displayed by the free drug. A last example that illustrates the advantages of the SLNs in cancer therapy is presented by indirubin, a hydrophobic anticancer agent ingredient in traditional Chinese medicine, which was loaded in SLNs and used against human glioblastoma cells [31]. The biological evaluation of these SLN-based formulations revealed a striking improvement of the anticancer effect of the hydrophobic drug.

### 2.3. Nanostructured Lipid Carriers (NLC)

**Figure 3:**
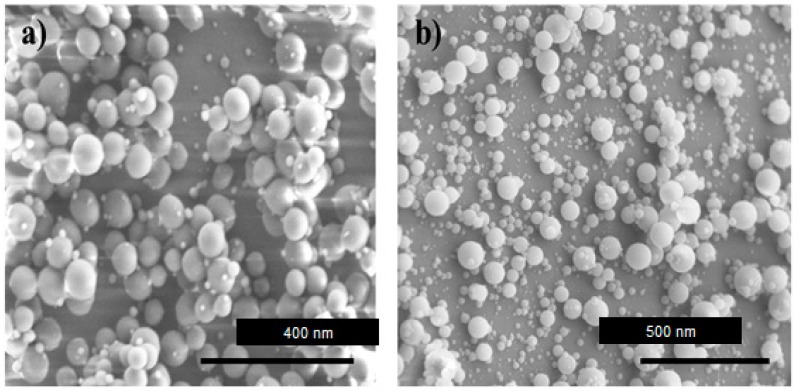
TEM images of nanostructured lipid carriers having (**a**) triestearin and (**b**) tripalmitin, as the main lipid component.

NLCs represent a second generation of lipid-based nanocarriers, developed from SLN, which comprise a combination of solid and liquid lipids. This system was developed in order to overcome the limitations of SLNs; hence, NLCs have higher drug loading capacity, and could also avoid drug expulsion during storage by avoiding lipid crystallization due to the presence of liquid lipids in the NLC formulation. While SLNs are composed of solid lipids, NLCs are a mixture of solid and liquid lipids, such as glyceryl tricaprylate, ethyl oleate, isopropyl myristate and glyceryl dioleate. The mean particle sizes are highly similar to SLNs, generally in the range of 10–1000 nm, and are affected by the nature of the containing lipids and the manufacturing process (Figure 3). The main advantages of these nanoparticles are that they can be loaded with hydrophilic and hydrophobic drugs, can be surface-modified, lend themselves to site-specific targeting, offer control of drug release, and exhibit low in vivo toxicity. However, there remain some disadvantages, such as drug expulsion after polymorphic transition of the lipid from the nanocarrier matrix in the storage period and low loading capacity [32].

Among the contributions reported in recent years, a first relevant example is presented by fluvastatin, which, when combined with lipoic acid and ellagic acid in a NLC, could be used as a candidate for prostate cancer therapy due to the effects of the combination with respect to cell death in comparison to free drugs [33]. Another interesting example was reported by Haron et al. [34], who used NLC in order to avoid the low bioavailability of lipophilic drugs such as thymoquinone (TQ). In that study, they developed a colloidal drug carrier TQ-NLC that displayed the anticancer effects on Hep3B liver cancer cells. Particularly interesting was the case of artesunate nanoparticles, modified by hyaluronic acid and cell-penetrating peptides, which showed very efficient results against cancer HepG2 cells due to their ability to effectively identify and penetrate the tumor cell membrane [35]. Also noteworthy the NLC loaded with orcinol-glycoside coated with PEG, a nanoformulation with potential for oral delivery, which showed anticancer activity against gastrointestinal cancer cell lines and hepatoma [36]. A final example in this section is the 6-Gingerol-loaded NLCs, which improved the water solubility and the oral bioavailability of the bioactive 6-gingerol, which presents poor water solubility [37].

## 3. Nanoparticles and Cancer

Nanoparticles constitute nano-sized carriers (5–200 nm) [38] whose variability and versatility can be seen in the wide range of applications that are being developed for them. The ability to control aspects such as shape, size, surface charge and composition at the atomic level, together with characteristics such as their biocompatibility or their ability to transport insoluble substances, makes them an excellent tool for the treatment of numerous diseases, including cancer [39,40]. Cancer is one of the most important diseases in the world, and represents one of the most important clinical challenges due to its high incidence rate (with an estimated 18.1 million new cases in 2018) [41], its variability and heterogeneity, with lung, breast, colorectal and prostate cancer being the most frequent [41,42]. Together with this, the particular aggressiveness of some kinds of cancers and the lack of definitive curative treatments, with 9.6 million deaths estimated for 2018 by GLOBOCAN [41,42], make cancer a major priority in current biomedical research. Currently, treatments for cancer are mainly focused on surgical resection, when feasible, and/or chemotherapy, radiation and hormonal therapies [43]. Chemotherapeutic agents commonly used have numerous side effects (insomnia, fatigue, cognitive impairment, nausea, vomiting, anemia, weight loss) that diminish the quality of life of patients and, in many cases, are not sufficient due to the high relapse rate, which results in low survival rates in most cases [43,44,45]. In this aspect, nanoparticles have emerged as a new opportunity, since they are able to be specifically addressed to a target, display a controlled release of their load, increase half-life time in blood plasma, decrease the side effects caused by chemotherapeutic agents, which include systemic toxicity, and decrease the off-target distribution, or improve the accumulation of the drug at the tumor site [40,46]. On the other hand, their biocompatibility allows them to be easily reabsorbed or eliminated by the organism. In addition, when targeting is passive, they can take advantage of the Enhanced Permeability and Retention Effect (EPR), by which NPs tend to accumulate at the tumor site [45].

Active targeting is one of the most promising applications for NPs, since they make it possible to take advantage of the specific characteristics and specific profile of each tumor niche to direct treatments to it by functionalizing the NPs with antibodies, tumor-specific antigens (TSA), microRNAs or siRNA, which, together with the properties of certain nanomaterials used in their synthesis, such as pH and/or temperature dependent degradation, response to light, magnetic or ultrasound stimuli, make it possible to release its load specifically at the tumor site, decreasing systemic toxicity enormously with respect to traditional treatment [38,46,47].

Currently, NPs have acquired a dual profile, being able to help in the diagnosis of disease in addition to granting targeted and specific therapy of disease (teragnosis) [38]. In addition, NPs could make it possible in the future to deliver personalized treatment for each patient, thus improving their response to treatment and their survival rate, striving towards achieving a complete cure [48].

## 4. Application of Lipid-Based Nanoparticles in Cancer Therapy

Lipid-Based NPs (LBNPs) constitute a broad and diverse group of nanoparticles that are particularly relevant in BreC treatment. However, despite their variety, liposomes are so extensively used due to their high degree of biocompatibility and their ability to encapsulate a wide range of cargos. LBNPs are currently being used in several trials, and some of them (e.g., Doxil^®^ or Abraxane^®^) have already been approved for BreC treatment [6,38]. This section examines the most significant advances made in recent years regarding the use of LBNPs in the treatment of the most frequent types of cancer. Table 1 summarizes the main clinical trials and the most recent in vivo and in vitro studies carried out in the most frequent cancers.

### 4.1. Gastrointestinal Cancer

#### 4.1.1. Gastric and Esophageal Cancer

Gastric cancer (GC) is the fifth most common cancer and the third most common cause of cancer-related death worldwide [41,42]. Only gastric cancer without lymph node metastasis can be treated with surgical resection alone. By contrast, advanced gastric cancer should be treated with combined chemotherapy, which can cause serious side effects. Currently, new therapies based on the use of nanoformulation are being developed to improve the patient’s response. Liposomes have been widely used in GC treatment, associated with molecules such as Arg-Gly-Asp peptide [49], SATB1 siRNA/CD44 antibodies [50], or forming DNA complexes [51]. Their application increased drug accumulation in tumor-bearing mice transplanted with SGC7901 cells with high expression of integrin α_5_β_1_ [49]. Additionally, liposomes demonstrated improved targeting precision and were able to silence gene expression of *SATB1* by approximately 80% in CD44^+^ GC initiating cells [50]. In addition, liposomes were able to recognize peritoneally disseminated GC MKN-45P cells, reducing their accumulation in the liver [51]. Preliminary trials with SLNs in GC [52] showed an enhanced activity of etoposide (VP16) in SGC-7901 cells, improving growth inhibition, producing cell arrest in G2/M phase (17.13%), and inducing mitochondria-involved apoptosis. Li et al. [53] designed a SLN for combined treatment with all-trans retinoic acid (ATRA) and sorafenib (both with a limited solubility), and miR-542-3p. This system increased the uptake of both anticancer drugs and produced a synergistic effect against MGC-803 cells. In vitro and in vivo evaluation of SLNs for the co-administration of PTX and tanespimycin (a heat shock protein 90 inhibitor) showed a significant inhibition of proliferation in SGC-7901, MKN-45 and AGS cells [54]. Jiang et al. [55] designed another co-delivery drug system by synthesizing NLCs loaded with VP16 and CUR, [56], which exhibited a significant reduction of IC50. As a final example, it is interesting to highlight the system designed by Qu et al. [57], comprising a prodrug of 5-FU and cisplatin co-encapsulated in a NLC and coated with hyaluronic acid. With this system, a synergistic effect between the drugs was observed when they were administered in ratios of 10:1 and 20:1 in BGC-823 cells, reducing tumor growth and reconstituting the body weight of mice bearing BGC-823 xenografts.

On the other hand, for esophageal cancer (EC), the seventh most frequent cancer worldwide [41,42], some new NPs have been assayed. Chang et al. [58] used the well-known rhenium-188 (^188^Re)-liposome in combination with radiotherapy to evaluate its effectiveness in BE-3 (esophageal adenocarcinoma) tumor-bearing mice. This system determined an increased tumor growth inhibition, with no alterations of WBC, hemogram profile or weight loss. Another interesting approach, developed by Feng et al. [59], was the use of PEG-LY294002 (autophagy inhibitor)-nanoliposome loaded with 5-FU. This nanoliposome made it possible to release the autophagy inhibitor more quickly, increasing the effect of 5-FU in a synergistic way, and achieving higher cell death rates in EC-9706 cells. In addition, this nanoliposome inhibited tumor growth in esophageal carcinoma OE-19 cells xenograft models.

#### 4.1.2. Colorectal Cancer

Colorectal cancer represents a serious health problem due to its high mortality (it is the second most common cause of cancer-related death) and the continuous increase in its incidence in recent years [41,42]. LBNPs represent a potential mechanism to improve the current treatment, especially in metastatic colon cancer in which chemotherapy (5-FU alone or in association with other drugs) or monoclonal antibodies (bevacizumab, trastuzumab, cetuximab) have a low efficacy. For example, a thermosensitive gel-mediated 5-FU microemulsion (ME) was able to increase the permeability and cell uptake of Caco-2, as well as its accumulation in rectal tissue in vivo in comparison with 5-FU thermosensitive gel [60]. Low et al. [61] designed a complex system based on Pickering emulsions (PE), composed of a magnetic cellulose nanocrystal loaded with CUR capable of releasing the drug in a controlled manner by exposure to an external magnetic field. This system decreased the growth of HCT116 cells in both monolayer and multicellular spheroids. In addition, Ektate et al. [62] used the lipopolysaccharide (LPS) from attenuated Salmonella bacteria loaded with DOX-thermosensitive liposomes and high-intensity focused ultrasonic waves to activate macrophages in the tumor environment. This system made it possible to improve the internalization of DOX through changes in membrane fluidity and to decrease the in vivo tumor growth. Functionalization of the liposomes has also been used to improve CRC treatment. Accordingly, Moghimipour et al. [63] used FoA to promote the internalization of 5-FU in CT-26 cells, significantly reducing its IC50 and decreasing tumor volume. Kaseem et al. [64] synthesized niosomes loaded with imatinib mesylate (IM), which was capable of reducing the IC50 of the free drug (~16-fold) in HCT-116 cells. Saber et al. [65] developed cisplatin-cubosomes that showed a reduction in the IC50 values of the free drug, as well as a synergistic effect by its co-administration with metformin in HCT-116 cells. In these assays, an increase in the caspase-3 activity following treatment was detected. Furthermore, Serini et al. [65] encapsulated omega-3 polyunsaturated fatty acids (docosahexaenoic and linoleic acid) in SLNs, demonstrating a growth inhibitory effect on HT-29 and HCT-116 cells. Interestingly, a complex system has been proposed to treat peritoneal metastases. This consists of oral administration of DOX-FoA-dextran-superparamagnetic iron oxide-loaded SLNs along with the application of high-frequency magnetic fields. As Shen et al. [66] demonstrated, this treatment was able to decrease the main tumor mass (15-fold) and to reduce the number and size of tumor nodules in the peritoneal cavity. Regarding NLCs, Negi et al. [67] designed a new formulation of hyaluronic acid-IRI-NLC capable of restoring the sensitivity of Colo-320 cells (with *MDR*-P-gp overexpression) to IRI by reducing the IC50 of the free drug 6 and 9.5 times at 72 and 96 h of exposure, respectively.

#### 4.1.3. Pancreatic Cancer

There are no feasible screening tests for early detection of pancreatic cancer (PaC). Therefore, PaC is often detected in advanced stages, when surgery cannot be applied. However, the failure of pancreatic cancer treatment is not only caused by the advanced state of the tumor (usually with metastases), but also by the difficulty the drugs have in penetrating deeply, given the nature of the tumor stroma. Nanotechnology offers some therapeutic strategies to improve the prognosis of these patients. Chirio et al. [68] prepared stearoyl chitosan-coated lipid NPs using the ME cold dilution technique, and loaded them with CUR. This system managed to increase the inhibition of cell growth (3 fold) for CUR (10μM) in PANC-1 cells. The efficacy of gemcitabine (first line treatment against PaC) was improved by using the γ-tocotrienol isomer of vitamin E and its encapsulation in a γ-T3/γ-T-mPEG 2000 core/corona nanoemulsion (NE). This association exhibited a higher antitumor activity than free gemcitabine in Bx-PC-3 cells and in gemcitabine-resistant PANC-1 cells [69]. Recently, Wood et al. [70] developed PEs composed of glycerol monostearate (shell), sorbitan monooleate (surfactant), and oseltamivir phosphate (OsP) as cargo, which made it possible to maintain a sustained release of OsP (30 days) in PANC-1 cells. Moreover, PEG-EF24 (a synthetic CUR analog)-liposomes inhibited the ability to form colonies of MIAPaCa and Pa03C cells in vitro, and showed a synergistic tumor growth inhibition when administered with gemcitabine in experiments in vivo using MIAPaCa xenografts models [71]. Wei et al. [72] developed human serum albumin-complexes associated with PTX and ellagic acid encapsulated in thermosensitive liposomes. This system increased the half-life time of PTX and its cellular uptake, and inhibited tumor growth in BxPC-3/HPaSteC-bearing mice when combined with heat. Finally, some of the recently developed therapeutic strategies have been tested in humans. In this vein, it is worth mentioning a phase III clinical trial evaluating the treatment of advanced PaC using nanoliposomal IRI (nal-IRI) [73]. This study was carried out in patients with prior gemcitabine-based therapy, and the regimen tested was nal-IRI in combination with 5-FU/leucovorin (nal-IRI+5-FU/LV) versus 5-FU alone. The combined treatment showed a higher overall survival, as well as a lower hazard ratio in patients with unfavorable prognosis [74].

#### 4.1.4. Liver Cancer

Liver cancer (LivC) therapies are often limited by poor drug physicochemical properties. In fact, chemotherapy and targeted drugs such as sorafenib induce a minimal impact in patient survival. In addition, radiotherapy is generally ineffective. Combining drugs with some nanoplatforms has been proposed as a strategy to increase overall treatment efficacy and patient survival. In this context, 5-FU-loaded cubosomes and PTX-loaded NLCs have been used in LivC treatment. On the one hand, 5-FU-loaded cubosomes prevent the drug from its rapid enzymatic degradation, increasing its accumulation in the liver. On the other hand, PTX-loaded NLCs improve the effect of the commercial formulation Intaxel^®^, benefitting its accumulation and permanence in plasma [75,76]. In addition, a system constituted by sorafenib and SPIONs co-loaded in SLNs has been developed as a dual treatment against HepG2 cells [77]. Furthermore, new nanoformulations are being developed and tested to increase the available antitumor strategies against LivC. For example, Lin et al. [78] and Qu et al. [79] developed two new MEs: (1) a CUR-loaded soybean-based ME, and (2) an ME based on Coix seed components (C-ME) modified with an octanoyl galactose ester to actively target the asialoglycoprotein receptor, overexpressed in hepatoma cells. The former was demonstrated to cause toxicity in HepG2 cells preferentially at low concentrations (15 μM), while the latter made it possible to increase both cellular internalization and cytotoxicity both in HepG2 cells and in HepG2 tumor xenograft-bearing mice. In addition, Hu et al. [80] designed a DOX and indocyanine green (ICG) co-loaded galactose functionalized PE for a combined treatment (photothermal and chemotherapeutic) against LivC. This strategy resulted in a complete inhibition of H22-tumor models when applied in combination with NIR laser irradiation. Finally, liposomal formulations like cantharidin-PEG-liposomes [81] or CUR-glycyrrhetinic acid-cationic liposomes [82] have shown excellent results in HepG2 and H-22 cells, respectively, increasing the inhibition of cell growth and inducing death by apoptosis both in vitro and in vivo. Furthermore, the liposomal formulation MRX34 (liposomal miR-34a mimic) associated with dexamethasone premedication has been demonstrated to be feasible, tolerable and able to induce antitumor activity in a phase I clinical trial in which most patients suffered from hepatocellular carcinoma [83].

### 4.2. Nervous System Cancer: Glioblastoma Multiforme

Glioblastoma multiforme (GBM) is the most aggressive and frequent type of malignant brain tumor (50% of glioma cases), with a very low 5-year survival rate (10%) [84]. The current treatment, based on surgical resection, temozolomide (TMZ) and radiation still implies low survival rates (15 months). Limitations to the current GBM treatment include the difficulty of resecting all of the tumor, the presence of protrusions infiltrating healthy brain tissues, and the “blood-brain barrier”, which limits the passage of drugs from the bloodstream into the brain. Some nanomaterials have demonstrated the ability to cross the blood–brain barrier (BBB) and to remain in GBM tissues, facilitating active targeting of the drug towards GBM tissues. For example, CUR is able to achieve an enhanced brain uptake by being encapsulated in a carrier. Accordingly, CUR-docosahexaenoic acid (DHA)-ME and CUR-NE were capable of boosting the effect of the drug in U-87MG from GBM [85,86]. The composite system based on transferrin-PFVYLI peptide (PFV)-liposomes co-loaded with DOX and Erlotinib, designed by Lakkadwala and Singh [87], were demonstrated to be able to increase drug uptake above 65% in U87 cells by means of clathrin-mediated endocytosis. In addition, this nanodrug was able to increase the transport of NPs through the endothelial barrier of a brain tumor model. Recently, a SLN functionalized with Angiopep-2 for an active targeting of GBM cells, and loaded with docetaxel, increased the average survival in intracranial GBM-bearing mice [88]. Notably, the NLC-based systems loaded with ferulic acid (FA) demonstrated their ability to improve its effect in U87MG cells [89]. Similarly, NLCs functionalized with lactoferrin and arginine–glycine–aspartic acid (RGD) peptide associated with TMZ and vincristine achieved a controlled release and a synergistic effect of both drugs in vivo [90]. On the other hand, TMZ treatment has also been improved by using NPs. For example, Papachristodoulou et al. [91] developed O^6^-(4-bromothenyl)guanine (O^6^BTG) derivative/N9-modified liposomes capable of inhibiting the expression of the *MGMT* gene in SMA-497 and ZH-161 cells. Furthermore, the intravenous application of O6BTG derivative liposomes and TMZ in a GBM intracranial model using SMA-497 cells resulted in a reduction of tumor growth and in an increase in survival rates in comparison with other treatments. In conclusion, the results of some clinical trials reinforce the potential application of these new therapeutic strategies in GBM. In fact, Myocet^®^, a non-PEG-DOX liposome, was used in pediatric patients with high-grade glioma (GBM, astrocytoma, oligodendroglioma or oligoastrocytoma) after TMZ-based chemotherapy (phase I clinical trial) [92]. With the same purpose, nanoliposomal IRI (nal-IRI) was used in adults (28–65 years) with high-grade glioma, making it possible to establish the maximum tolerated dose. This phase I clinical trial demonstrated a prolonged permanence of the drug in blood, slowing down its conversion into SN-38 (active metabolite) [93].

### 4.3. Lung Cancer

Lung cancer (LuC) is the most common cancer and the primary cause of cancer-related death worldwide in both men and women [41,42]. Despite chemotherapy and radiotherapy being successful in lung cancer, most patients unfortunately develop a recurrence of the disease that is more resistant to subsequent therapy. Therefore, a new therapeutic strategy is required to improve the prognosis of this type of cancer. In recent years, significant advances have been made in the development and application of nanotechnology to the diagnosis and therapy of this type of cancer, including the use of LBNPs. For example, bromo-noscapine NE [94], lipophilic diferuloylmethane NE [95], CUR-water-Free-NE [96] and docetaxel-NE [97] showed increased antitumor activity in A-549 cells. The nebulized docetaxel-NE was even more effective against tumor cells (A549 cells) than against normal cells (MRC-5 cells) [97]. Moreover, an RGD peptide-lycobetaine-oleic acid-PEG-NE increased survival and reduced tumor volume in vivo, in comparison with each component separately [98]. On the other hand, Shi et al. [99] developed an SLN-PTX associated with miRNA-34a which was applied in B16F10 (CD44+) lung metastasis-bearing mice. These authors demonstrated a synergistic effect of both agents. Similarly, transferrin-PEG-hydrazone-SLN was used to co-administer baicalin and docetaxel, obtaining a strong synergistic effect in A549 tumor-bearing mice [100]. Liang et al. [101] developed a functionalized system with a glucose receptor-targeting ligand for the co-encapsulation of PTX and gemcitabine (GEM). These authors demonstrated that GEM/PTX in a ratio of 3:1 induced a strong synergistic effect in A549. Finally, some clinical trials showed promising results in lung cancer. For example, a phase III clinical trial combined intratumoral PTX liposome administration along with systemic carboplatin and gemcitabine in patients with unresectable non-small cell lung cancer. The results obtained with this combination increased the resection rate with an acceptable toxicity [102].

### 4.4. Breast Cancer

Breast cancer, the primary cause of cancer-related death in women [41,42], is currently undergoing significant changes due to the development of NPs, particularly in relation to the treatment of advanced cancer stages. NEs loaded with DOX and bromotetratrandrine (W198, P-glycoprotein (P-gp) inhibitor) were tested in the resistant MCF-7/ADR cell line. This resulted in a higher cellular uptake and accumulation of DOX in the tumor tissue. Interestingly, DOX showed a decrease in gastrointestinal and cardiac toxicity [103]. In addition, formulations based on DOX-liposomes have been tested in clinical trials. Recently, PEG-DOX liposomes (PLD) in combination with lapatinib were used in HER2-positive BreC patients (phase Ib) to determine the most appropriate combination of both treatments at the maximum tolerated dose [104]. Moreover, a phase III clinical trial co-administering Myocet^©^ with cyclophosphamide (CM) or vinorelbine (MV) in patients with metastatic BreC has also been developed [105].

On the other hand, niosomes have become one of the most interesting LBNPs in BreC treatment, which has led to the development of various systems to facilitate the vehiculization of gingerol [106], tamoxifen citrate (TXC) [107,108], carboplatin [109], silibinin [110,111], lawsone [112], mitoxantrone [113] or TQ [114]. Accordingly, Shaker et al. [108] synthesized a TXC-niosome capable of increasing drug uptake in MCF-7 cells and reducing tumor volume in Ehrlich carcinoma model in vivo. Rajput et al. [114] designed a complex system based on TQ-gold-niosomes loaded with Akt-siRNA capable of improving the anti-tumor effect of TQ in TXC-resistant and Akt-overexpressing MCF-7 cells. Other systems have also been tested in BreC treatment, such as archaeosomes synthesized with a certain ratio of paclitaxel [115] and cubosomes functionalized with FoA-P407 and loaded with etoposide [116].

Other LBNPs used in BreC research include SLNs. Yu et al. [117] proposed a system for the combined administration of PTX and functionalized DNA with a pH-sensitive ligand. This system was able to reduce tumor volume in vivo, decreasing PTX accumulation in other organs. Moreover, Garg et al. [118] synthesized a fucose-methotrexate SLN which showed a preferential accumulation in the tumor tissue only 2 h after its administration, in comparison with free methotrexate, which tends to accumulate in the kidneys, liver and spleen. Finally, Liang et al. [119] and Li et al. [120] designed two new NLC systems for BreC treatment. Liang et al. [119] used a NLC functionalized with a HER2 aptamer and loaded with an ATP aptamer bound to EGCG and protamine sulfate to inhibit the growth of SK-BR-3 cells (HER2 receptor overexpressed). On the other hand, Li et al. [120] developed a NLC co-loaded with DOX and lapachone, which made it possible to increase the DOX concentration in tumor tissues in comparison with the free DOX in MCF-7/ADR tumor xenograft-bearing mice.

### 4.5. Prostate Cancer

Currently, NEs, liposomes and solid-lipid NPs (SLNs) are the main LBNPs being tested as new therapeutic strategies in prostate cancer (PrC). Recently, Ahmad et al. [121] developed an oil-in-water NE loaded with a taxoid prodrug associated with an omega-3 fatty acid. This NE was capable of reducing the taxoid IC50 of PPT2 cells 12-fold, allowing a greater tumor volume reduction than Abraxane™ in tumor-bearing mice. Similar antitumor benefits were observed by using NE loaded with catwechin extract (flavanols with anticancer properties) in PC-3 cells [122]. With respect to liposomes, 22Rv1 PrC cells were exposed to PEG-folate-targeted-oleuropein-liposomes [123]. These nanoplatforms increased the level of 22Rv1 cell apoptosis, the oleuropein bioavailability and the survival in in vivo models [123]. In addition, NPs were developed by Hua et al. [124] that consisted of a multifunctional liposome loaded with docetaxel and gold nanorod, showing 100% inhibition of PrC cell growth by combining the system with the application of a laser.

## 5. Lipid-Based Nanoparticles, Drug Resistance and Epithelial-Mesenchymal Transition

**Figure 4:**
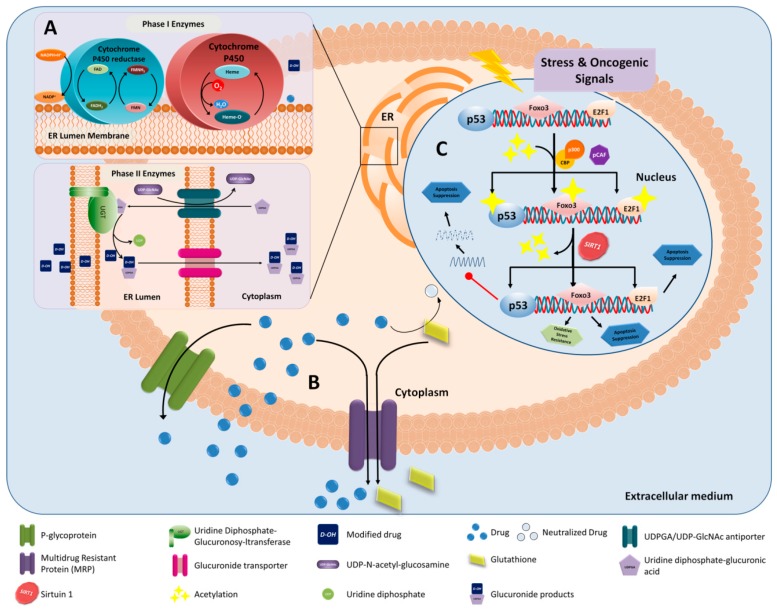
Multidrug resistance mechanisms. Altered drug metabolism (**A**), ABC transporters increasing drug efflux (**B**) and stress and oncogenic signals induce the acetylation of transcription factors (e.g., Foxo3, E2F1) and tumor suppressor genes (*p53*) (**C**) while enzymes such as SIRT1 cause deacetylation, thus suppressing apoptotic processes and conferring resistance to oxidative stress.

Drug resistance (DR) is an essential aspect that limits the effectiveness and application of cancer treatment [126]. A distinction should be established between two types of DR: (1) primary DR, which occurs before applying any treatment; and (2) acquired or multiple drug resistance (MDR), which occurs when a treatment produces resistance to another set of drugs [127]. Some of the most important mechanisms of resistance in cancer (Figure 4) imply enzymes involved in drug metabolism (e.g., cytochrome P450, glutathione S-transferase) and membrane transporters which modify the influx and efflux of drugs (ABC transporters) [126]. The possibility of blocking the mechanism of resistance by using nanoplatforms is a promising therapeutic strategy in cancer. Baek et al. [128] developed a folate-conjugated SLN capable of differentially and sequentially releasing two co-encapsulated antitumor drugs, CUR and PTX, so that CUR, capable of inhibiting P-gp, starts being released faster than PTX. In this way, the inhibition of P-gp in MCF-7/ADR BreC-resistant cells is ensured, allowing the accumulation of PTX inside. In addition, other mechanisms have been involved in MDR, including DNA damage repair (increased repair), long non-coding RNAs, epigenetics (DNA methylation, histone modification, microRNAs) and oncogenes (*p53*, *MALT1*, *KRAS*, *HGAL*, *ERBB2*, *PIK3CA*, *AKT*) [126,129]. In this context, Zhao et al. [130] synthesized hyaluronic acid/DOTAP liposomes core/shell-NPs loaded with polymetformin, a conjugate of dicyandiamide with polyethylenimine, capable of harboring vascular endothelial growth factor (VEGF) siRNA. This system achieved 95% VEGF silencing in LuC-bearing mice increasing apoptosis (up to 40%) in the tumor mass. However, the effectiveness of this nanoplatform did not come only from VEGF siRNA. Accordingly, the studies carried out without the siRNA demonstrated an antitumor activity mediated by polymetformin, which inhibited mTOR and activated AMPK pathways, responsible for the DNA damage response and tumor suppressor activity, respectively.

Finally, the epithelial-mesenchymal transition (EMT), by which epithelial cells lose typical markers of differentiation (mainly E-cadherin) and begin to express markers of mesenchymal cell differentiation (N-cadherin), may be relevant in cancer resistance. This transition has been linked to processes such as cancer metastasis. Therefore, it is suspected that EMT could induce cancer stem cell (CSC) characteristics by increasing MDR phenomena [126,131]. In this regard, Li et al. [132] designed LBNPs loaded with diacidic norcantharidin and functionalized with the RGD peptide that were capable of binding to the cellular membrane protein-integrin α5 and restoring the expression of E-cadherin. This expression was associated with a better prognosis in triple negative BreC xenograft models.

## 6. Conclusions

LBNPs constitute a diverse and extensive group that has been used in the treatment of numerous pathologies, mainly in cancer. Liposomes are the most widely used LBNPs, due to their great biocompatibility and versatility; however, recently, SLNs and NLCs have been gaining great attention. Even so, research is not focused solely on these NPs, and there are many publications focused on new strategies using LBNPs to treat different types of cancers. Some of these have even taken the leap to the next level and have begun their path in clinical trials, and what is more, some examples, such as Doxil^®^, Abraxane^®^, nal-IRI and Myocet^®^, have been approved in clinic for the treatment of cancer. This makes LBNPs a very promising group for the treatment of cancer in the future.

## Figures and Tables

**Table 1:** Lipid-based nanoparticles used in cancer treatment: clinical trials and in vivo and in vitro studies in relation to some of the most frequent cancers.

Cancer Type	NP Type	Target	Drug or Cargo	SS	Detection Method	Combined Therapy	Status	Reference
Gastric	Liposome	Integrin α5β1	RGD Peptide & ICG	PEG	CoM, FC & IVV imaging		IVT + IVV	[49]
	Liposome	SATB1	SATB1 siRNA	CD44 antibody	FC		IVT	[50]
	Liposome	Affinity ligand	Plasmid DNA & Tumor-homing peptides	PEG	FC& IVV imaging		IVT + IVV	[51]
	SLN	TopII	Etoposide (VP16)				IVT	[52]
	SLN	AEG-1, Ras/Raf/Mek/Erk cascade pathway & ReA receptors	miR-542-3p & Sorafenib	PEG		All-trans ReA	IVT	[53]
	SLN	Tubulin & Hsp90	PTX			17-AAG	IVT + IVV	[54]
	NLC	TopII	Etoposide (VP16)				IVT + IVV	[55]
	NLC	TopII	Etoposide (VP16)			CUR	IVT + IVV	[56]
	NLC	Thymidylate synthase	5-FU-stearic acid prodrug	Hyaluronic acid		Cisplatin	IVT + IVV	[57]
Esophageal	Liposome		Rhenium 188		NanoSPECT/CT scanner system		IVT + IVV	[58]
	Liposome	Thymidylate synthase	LY294002	PEG		5-FU	IVT + IVV	[59]
Colorectal	ME	Thymidylate synthase	5-FU				IVT + IVV	[60]
	PE		CUR				IVT	[61]
	Liposome	TopII	DOX	attenuated Salmonella		HIFU	IVT + IVV	[62]
	Liposome	Thymidylate synthase	5-FU	FoA			IVT + IVV	[63]
	Niosome	Protein-tyrosine kinase	IM				IVT	[64]
	Cubosome		Cisplatin			Metformin	IVT	[125]
	SLN		Omega-3 PUFA—DHA & Linoleic acid (LNA)				IVT	[65]
	SLN	TopII	DOX	FoA & Dextran		SPIONs/high-frequency magnetic field (HFMF)	IVT + IVV	[66]
	NLC	TopI	IRI	Hyaluronic acid			IVT	[67]
Pancreatic	ME		Cur	Stearoyl chitosan	Optical microscopy SEM		IVT + IVV	[68]
	NE		Gemcitabine			γ-tocotrienol isomer of vitamin E	IVT	[69]
	PE	Neu1	OsP				IVT	[70]
	Liposome	NF-kappaB	EF24	PEG	TEM	Gemcitabine	IVT + IVV	[71]
	Liposome		HSA-PTX & HSA-Ellagic acid		Inverted FM		IVT + IVV	[72]
	Liposome		nal-IRI & 5-FU/leucovorin				CT Phase III	[74]
Liver	ME		Cur		FM		IVT	[78]
	ME		Coix seed components	Octanoyl galactose ester	In vivo near-infrared imaging system		IVT + IVV	[79]
	PE	TopII	DOX & ICG	Galactose	NIR fluorescence imaging	NIR laser irradiation	IVT + IVV	[80]
	Liposome		Cantharidin	PEG			IVT + IVV	[81]
	Liposome		Cur	Glycyrrhetinic acid			IVT + IVV	[82]
	Liposome			miR-34a mimic			CT—Phase 1	[83]
	Cubosome	Thymidylate synthase	5-FU				IVT + IVV	[76]
	SLN		Sorafenib			SPIONs	IVT	[77]
	NLC	Tubulin	PTX				IVT + IVV	[75]
GBM	ME		Cur			DHA-rich oil	IVT + IVV	[86]
	NE		Cur				IVT + IVV	[85]
	Liposome	Tf receptors (TfR)	DOX	Tf & PFV		Erlotinib	IVT	[87]
	Liposome	*MGMT* gene	O^6^BTG derivative		LIFU			[91]
	Liposome		Myocet^®^ (DOX)				CT—Phase I	[92]
	Liposome	TopI	nal-IRI				CT—Phase I	[93]
	SLN	LRP-1	Docetaxel	Angiopep-2	Real time fluorescence imaging		IVT + IVV	[88]
	NLC		Ferulic acid				IVT	[89]
	NLC		TMZ	Lactoferrin & RGD peptide		Vincristine	IVT + IVV	[90]
Lung	NE		9-bromo-noscapine	Spray dried lactose			IVT + IVV	[94]
	NE		Lipophilic diferuloylmethane				IVT + IVV	[95]
	NE		Cur	Tween80 & LipodS75			IVT + IVV	[96]
	NE	Tubulin	Docetaxel				IVT	[97]
	NE		Lycobetaine & oleic acid (OA)	PEG-lecithin & nRGD peptide			IVT + IVV	[98]
	Liposome	Tubulin	PTX			Carboplatin & Gemcitabine	CT Phase III	[102]
	SLN	Tubulin	PTX	miR-34a	FC& CoM		IVT + IVV	[99]
	SLN	Tubulin & Tf receptors (TfR)	Docetaxel & Baicalin	PEG, Tf & Hydrazone			IVT + IVV	[100]
	NLC	Tubulin & Glucose receptor	Gemcitabine & PTX	Glucose receptor-targeting ligand	FC& CoM		IVT	[101]
Breast	NE	TopII& P-gp	DOX & W198		Whole body fluorescence imaging		IVT + IVV	[103]
	Liposome		DOX	PEG		Lapatinib	CT Phase Ib	[104]
	Liposome		Myocet^©^			Cyclophosphamide (MC) or vinorelbine (MV)	CT Phase III	[105]
	Niosome		Tamoxifen citrate (TXC)				IVT + IVV	[108]
	Niosome		TQ		Fluorescence Imaging & NIR	Akt-siRNA	IVT + IVV	[114]
	Archaeosomes		PTX				IVT	[115]
	Cubosomes	TopII	Etoposide (VP16)	FoA-P407			IVT + IVV	[116]
	SLN		PTX & DNA	Hyaluronic acid			IVT + IVV	[117]
	SLN		Methotrexate	Fucose			IVT + IVV	[118]
	NLC	HER2+	ATP aptamer-EGCG-protamine sulfate	HER2 aptamer			IVT + IVV	[119]
	NLC	TopII & NQO-1	Lapachone & DOX		Confocal laser scanning microscopy		IVT + IVV	[120]
Prostate	NE		Taxoid prodrug	Omega-3 fatty acid	CoM		IVT + IVV	[121]
	NE		Catechin extract		TEM		IVT	[122]
	Liposome		Oleuropein	PEG	CoM		IVT + IVV	[123]
	Liposome	LRP-1	Docetaxel	PEG	FM		IVT + IVV	[124]

Status: IVT—In vitro; IVV—In vivo; CT—Clinical Trial. Confocal Microscopy—CoM; Curcumin-CUR; Indocyanine green—ICG; Flow cytometry—FC; Fluorescent microscopy—FM; Folic Acid—FoA; microRNA-34a—miR-34a; Lipoprotein receptor related protein 1—LRP-1; Retinoic Acid—ReA; Surface functionalization—SS; Transferrin—Tf; Topoisomerase I—TopI; Topoisomerase II—TopII.
